# Three new species of the soil centipede genus *Californiphilus* (Geophilomorpha, Himantariidae) from central and southwest China

**DOI:** 10.3897/BDJ.14.e199879

**Published:** 2026-07-07

**Authors:** Chenlu Yang, Chunxue You, Chao Jiang

**Affiliations:** 1 Tianjin Key Laboratory of Agricultural Animal Breeding and Healthy Husbandry, College of Animal Science and Veterinary Medicine, Tianjin Agricultural University, 300392, Tianjin, China Tianjin Key Laboratory of Agricultural Animal Breeding and Healthy Husbandry, College of Animal Science and Veterinary Medicine, Tianjin Agricultural University, 300392 Tianjin China; 2 State Key Laboratory for Quality Ensurance and Sustainable Use of Dao-di Herbs, National Resource Center for Chinese Materia Medica, China Academy of Chinese Medical Sciences, 100700, Beijing, China State Key Laboratory for Quality Ensurance and Sustainable Use of Dao-di Herbs, National Resource Center for Chinese Materia Medica, China Academy of Chinese Medical Sciences, 100700 Beijing China

**Keywords:** China, distribution, key, morphology

## Abstract

**Background:**

Three new species of the soil centipede genus *Californiphilus* Verhoeff, 1938 (Geophilomorpha, Himantariidae) are described from central and southwest China: *C. cryptoporus* Yang, You & Jiang, **sp. nov**. from Jiangxi Province, *C. hubeiensis* Yang, You & Jiang, **sp. nov**. from Hubei and *C. nanus* Yang, You & Jiang, **sp. nov**. from Hubei, Hunan and Chongqing. Detailed morphological descriptions, illustrations and a key to the species of the genus are provided.

**New information:**

In this study, three new species of *Californiphilus* are described from central and southern China. This contribution increases the documented diversity of the genus in China and provides new morphological data.

## Introduction

The family Himantariidae Bollman, 1893 comprises a morphologically distinctive lineage of geophilomorphs, characterised by a short head, stout antennae, mandible with a single dentate lamella and several pectinate lamellae and a relatively large number of body segments (Bonato et al. 2011). Within Himantariidae, the genus *Californiphilus* Verhoeff, 1938 is a geographically distinct lineage with a discontinuous trans-Pacific distribution. The genus currently includes three known species, *C. michelbacheri* Verhoeff, 1938 from California, USA, *C. mexicanus* Attems, 1947 from Mexico and *C. japonicus* (Takakuwa 1935), which is distributed in Japan, the Korean Peninsula and China (Takakuwa 1935, Verhoeff 1938, Takakuwa 1940, Attems 1947, Takakuwa and Takashima 1949). Previously, *C. japonicus* had been recorded from several localities in China, including Dalian (Liaoning), Shanxi and Shengzhou (Zhejiang) (Takashima 1949, Takakuwa and Takashima 1949, Chamberlin and Wang 1952), although broader collection records remain scarce.

In China, *C. japonicus* has been recorded from a few scattered localities (Takakuwa 1940, Chamberlin and Wang 1952), but the diversity and distribution of the genus in the country remain poorly understood.

Recent field surveys in central and southern China yielded a series of *Californiphilus* specimens that differ consistently from all known congeners in several morphological characters. In this study, we describe three new species, provide a morphological comparison with all known species of the genus and present an updated identification key.

## Materials and methods

Specimens were collected between 2019 and 2025 by hand-collected rocks, leaf litter and soil using tweezers. No fixed quantitative sampling scheme was applied; instead, specimens were gathered randomly across various localities and microhabitats to maximise morphological diversity. Materials are preserved in 75% ethanol and deposited at the National Resource Center for Chinese Materia Medica, China Academy of Chinese Medical Sciences, Beijing, China (**CMMI**).

Specimens were dissected and the cephalic capsules, mandibles and maxillary complexes were mounted on temporary slides with 75% ethanol. To examine the coxal pores, samples were cleared in 75% lactic acid for approximately 5–10 hours until the internal structures became visible under a light microscope. Morphological examination and imaging were carried out using a Leica M205 FCA stereomicroscope and an Olympus BX51 microscope. Photographs were converted into hand-drawn illustrations with SKETCHBOOK 6.2.3.

Morphological terminology for external anatomy follows Bonato et al. (2010).

## Taxon treatments

### Californiphilus cryptoporus

Yang, You & Jiang sp. nov.

F709A520-A415-5423-92DF-B5729271061B

9B935BAF-30E4-43A8-8E57-44A2448B85F6

#### Materials

- **Type status:** Holotype. **Occurrence:** catalogNumber: 20231021005D; recordedBy: Chao Jiang; individualCount: 1; sex: male; lifeStage: adult; occurrenceID: D13E59AC-23DA-56D4-B006-6C364AD17B4A; **Taxon:** scientificName: Californiphilus cryptoporus; **Location:** country: China; stateProvince: Jiangxi; locality: Longxihe National Wetland Park; verbatimElevation: 40 m; verbatimCoordinates: 28.0447°N, 115.5188°E; **Event:** eventDate: 21.x.2023; **Record Level:** language: en; institutionCode: CMMI
- **Type status:** Paratype. **Occurrence:** catalogNumber: 20191015007; recordedBy: Chao Jiang; individualCount: 1; sex: male; lifeStage: adult; occurrenceID: 268A348C-613C-5486-BBC0-C3277AC69D50; **Taxon:** scientificName: Californiphilus cryptoporus; **Location:** country: China; stateProvince: Jiangxi; locality: Yaodu Park; verbatimElevation: 30 m; verbatimCoordinates: 28.0601°N, 115.5403°E; **Event:** eventDate: 15.x.2019; **Record Level:** language: en; institutionCode: CMMI
- **Type status:** Paratype. **Occurrence:** catalogNumber: 20191015008; recordedBy: Chao Jiang; individualCount: 1; sex: female; lifeStage: adult; occurrenceID: 6E4092C4-8D4F-551B-845E-3C8DAD2EA188; **Taxon:** scientificName: Californiphilus cryptoporus; **Location:** country: China; stateProvince: Jiangxi; locality: Yaodu Park; verbatimElevation: 30 m; verbatimCoordinates: 28.0601°N, 115.5403°E; **Event:** eventDate: 15.x.2019; **Record Level:** language: en; institutionCode: CMMI
- **Type status:** Paratype. **Occurrence:** catalogNumber: 20191016030; recordedBy: Chao Jiang; individualCount: 1; sex: female; lifeStage: adult; occurrenceID: C7884C7B-A2C7-531F-8A57-3F6BA26F892F; **Taxon:** scientificName: Californiphilus cryptoporus; **Location:** country: China; stateProvince: Jiangxi; locality: Yaodu Park; verbatimElevation: 30 m; verbatimCoordinates: 28.0601°N, 115.5403°E; **Event:** eventDate: 16.x.2019; **Record Level:** language: en; institutionCode: CMMI
- **Type status:** Paratype. **Occurrence:** catalogNumber: 20191016031; recordedBy: Chao Jiang; individualCount: 1; sex: female; lifeStage: adult; occurrenceID: CC577EAB-436D-522F-BC09-FB2E53AD37CA; **Taxon:** scientificName: Californiphilus cryptoporus; **Location:** stateProvince: Jiangxi; locality: Yaodu Park; verbatimElevation: 30 m; verbatimCoordinates: 28.0601°N, 115.5403°E; **Event:** eventDate: 16.x.2019; **Record Level:** language: en; institutionCode: CMMI
- **Type status:** Paratype. **Occurrence:** catalogNumber: 20191016032; recordedBy: Chao Jiang; individualCount: 1; sex: female; lifeStage: adult; occurrenceID: 9A3098FC-2A54-5C27-831A-C3F0CC35BA59; **Taxon:** scientificName: Californiphilus cryptoporus; **Location:** stateProvince: Jiangxi; locality: Yaodu Park; verbatimElevation: 30 m; verbatimCoordinates: 28.0601°N, 115.5403°E; **Event:** eventDate: 16.x.2019; **Record Level:** language: en; institutionCode: CMMI
- **Type status:** Paratype. **Occurrence:** catalogNumber: 20260329025; recordedBy: Yuan Xiong & Qing Li; individualCount: 1; sex: female; lifeStage: adult; occurrenceID: 35F3B0AF-67C1-5452-ABC7-296B07C28112; **Taxon:** scientificName: Californiphilus cryptoporus; **Location:** stateProvince: Henan; county: Fangcheng; locality: Dachengshan Scenic Area; verbatimElevation: 260 m; verbatimCoordinates: 33.1569°N, 113.1468°E; **Event:** eventDate: 29.iii.2026; **Record Level:** language: en; institutionCode: CMMI

#### Description

**General features**. Body length of males 59–61 mm, of females 30–63 mm, with 71–77 (females: 73–77; males: 71) leg-bearing segments. Body narrowing forwards and towards posterior tip. Colour (in 75% ethanol) brown; forcipules darker.

**Figure 1.**
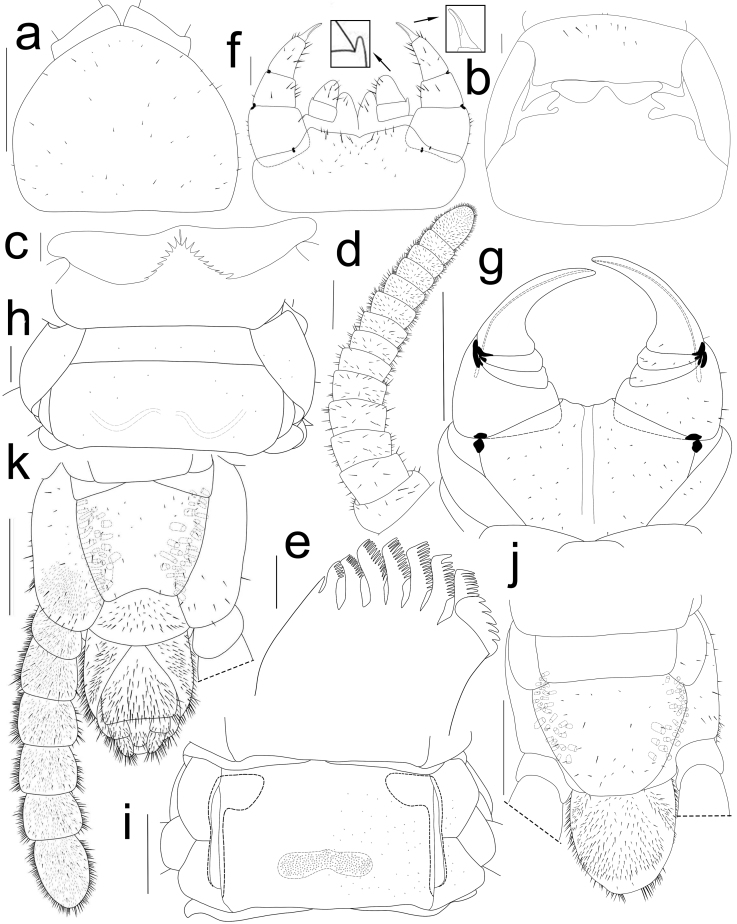
*Californiphilus cryptoporus* sp. nov. **a** Cephalic capsule, dorsal; **b** Anterior part of cephalic capsule, ventral (forcipules, maxillae and mandibles removed); **c** Labrum, ventral view; **d** Left antenna, ventral; **e** Mandible; **f** Maxillary complex, ventral; **g** Forcipular segment; **h** Tergite of leg-bearing segment, dorsal; **i** Sternum of the middle trunk segments, ventral (setae on right part omitted); **j** Posterior end of body in adult male, dorsal (ultimate leg omitted); **k** Posterior end of body in adult male, ventral (left leg omitted), ventral. Specimen: male, holotype (**a–k**). Scale bars: 500 μm (**a, b, d, g–k**); 100 μm (**f**); 50 μm (**c**); 20 μm (**e**).

**Cephalic capsule** (Fig. 1a) widest posteriorly, being a little narrowed cephalic to anterior corners, the border in front of corners triangular, ca. 1.6× as wide as long, lateral margins slightly convex, converging forwards, posterior margin almost straight, transverse suture absent, setae randomly scattered.

**Clypeus and labrum** (Fig. 1b and c). Clypeus anterior part with a few randomly scattered setae, 1+1 post-antennal setae aligned in two transverse rows on the anterior part of the clypeus, the first row bearing ca. 6 setae and the second row ca. 3 setae. Without areolate part. Labrum median arc deeply embayed, with 8–10 denticles on each side; entirely demarcated from clypeus by clypeolabral suture.

**Antennae** (Fig. 1d) composed of 14 articles, attenuated distally. Basal articles 1–4 slightly wider than long or sub-quadrate, with sparse long setae. From ca. 7 article onwards, setae much shorter and denser. Ultimate article distinctly elongate, ca. 1.5× as long as wide, 2× length of penultimate article.

**Mandible** (Fig. 1e) with a deeply pigmented strong dentate lamella, long axis slightly angled to distal mandibular edge, occupying ca. 1/4 width of distal edge, with seven well-developed pectinate lamellae, a rudiment pectinate lamella. Pectinate lamellae each with 6–14 comb teeth, dentate lamella with 6–7 denticules.

**First maxillae** (Fig. 1f) coxosternite of first maxillae divided; telopodite biarticulated, with the basal article cylindrical and the distal article conical; telopodital lappet present, short and pointed, ca. 1/8 the length of the basal article. Each side of the coxal process with 2–3 setae apically and the distal article of the telopodite with 5–7 setae each side on its anterior part.

**Second maxillae** (Fig. 1f) coxosternite with a slight median notch, anterior part with sparse scattered setae. Telopodite three-segmented: article 1 with short and sparse setae scattered on the outer margin; article 2 and article 3 with longer setae concentrated on the inner margin and the anterior part. Claws slightly spatulate, without basal spine.

**Forcipular segment** (Fig. 1g) forcipular tergite sub-trapezoid; ca. 3.0× as wide as long. Coxosternite exposed part ca. 2.0× as wide as long, trochanteroprefemur ca. 1.2× as wide as long; interior margin moderately projecting with respect to its condyles; anterior border approximately straight medially; coxopleural sutures strongly converging backwards; chitin-lines complete, reaching the coxosternal condyle. Tarsungulum large and pointed, not reaching cephalic capsule anterior margin when closed. All articles without denticles. Poison gland calyx linear, ca. 5.5× as long as wide, situated in anterior 1/4 of trochanteroprefemur. Entire forcipular segment without denticles.

**Leg-bearing segment** (Fig. 1h and i) Tergites sub-trapezoid, ca. 2.0× as wide as long, with short sparse setae, without paramedian sulci and preparatergites. Metasternites sub-trapezoid, ca. 1.5× as wide as long, with short sparse scattered setae. Lateral gutter and virguliform fossa begin on segments 23–26 and extend to segments 34–39. Posterior reniform ventral pore-fields present in all sternites from 1 to penultimate, pore-fields posteriorly slightly embayed. Pore-fields on sternite 1 distinctly smaller than those on succeeding sternites, sub-oval.

**Ultimate leg-bearing segment** (Fig. 1j and k). Pretergite ca. 3.2× as wide as long on exposed part. Metatergite ca. 1.3× as long as wide; sub-trapezoid. converging backwards; posterior margin ca. 0.6× as wide as anterior margin; with sparse setae of various sizes. Metasternite sub-trapeziform, ca. 1.3× as long as wide; narrowing posteriorly, posterior margin concave anteriorly, without median sulci; coxal pores 28–30 on each side, opening underside of the metasternite of the ultimate leg-bearing segments, distributed along the lateral margins of metasternite.

Ultimate leg ca. 1.1× as long as penultimate leg, without claw; male distinctly swollen, ventral and lateral sides with dense setae, female short and slender, with sparse setae.

**Postpedal segments** (Fig. 1j and k). Female gonopods unarticulated, posterior margin slightly concave (Fig. 6a), uniformly with sparse setae; male gonopods bi-articulate, with dense setae, penis conical. Anal pores absent.

#### Diagnosis

Body length reaching at least 30 mm; number of leg-bearing segments usually 71–77; Lateral gutter and virguliform fossa begin on segments 23–26 and extend to segments 34–39. Coxal pores numerous, opening underside of the metasternite of the ultimate leg-bearing segment, distributed along the lateral margins of metasternite.

#### Etymology

Greek: crypto- = hidden; Latin: porus = pore. The specific epithet refers to nearly all coxal pores covered by the lateral margins of the metasternite and metatergite. We suggest its Chinese common name as “隐孔地蜈蚣”.

#### Distribution

*C. cryptoporus* is known from three localities in Jiangxi and Henan, China (Longxihe Wetland Park, Yaodu Park and Dachengshan Scenic Area), all in the Dabie Mountain range, within subtropical evergreen broad-leaved forests, mainly in secondary forest patches in urban parks.

#### Notes

This species differs from other species of *Californiphilus* in that the openings of the coxal pores are almost entirely covered by the lateral margins of the ultimate tergite and ultimate sternite, becoming visible only after clearing with lactic acid or glycerol (Fig. 5b, Fig. 1j and k). In contrast, in the other species of this genus, the openings are not covered by these margins; instead, they are exposed and open directly into distinct pouches, located along the margins of the ultimate sternite and tergite.

### Californiphilus hubeiensis

Yang, You & Jiang sp. nov.

AA97AA30-CD1A-531F-820E-4BEE01F8B6EC

7455C7EC-B7EE-4162-AA20-E99C3241C050

#### Materials

- **Type status:** Holotype. **Occurrence:** catalogNumber: 20230427003D; recordedBy: Tenyun Chen, Yanyang Pan and Jiabo Fan; individualCount: 1; sex: male; lifeStage: adult; occurrenceID: 2D196A47-6F70-51D1-8534-75C147AE5ABF; **Taxon:** scientificName: Californiphilus hubeiensis; **Location:** country: China; stateProvince: Hubei; locality: Wuhan, Ma’anshan; verbatimElevation: 110 m; verbatimCoordinates: 30.5146°N, 114.4394°E; **Event:** eventDate: 27.iv.2023; **Record Level:** language: en; institutionCode: CMMI
- **Type status:** Paratype. **Occurrence:** catalogNumber: 20230427001D; recordedBy: Tenyun Chen, Yanyang Pan and Jiabo Fan; individualCount: 1; sex: male; lifeStage: adult; occurrenceID: F767E51B-D755-56C2-A14E-FDA956DCDAF5; **Taxon:** scientificName: Californiphilus hubeiensis; **Location:** country: China; stateProvince: Hubei; locality: Tonghu Provincial Wetland Park; verbatimElevation: 20 m; verbatimCoordinates: 30.3822°N, 113.9501°E; **Event:** eventDate: 27.iv.2023; **Record Level:** language: en; institutionCode: CMMI
- **Type status:** Paratype. **Occurrence:** catalogNumber: 20190402009; recordedBy: Chao Jiang; individualCount: 1; sex: male; lifeStage: adult; occurrenceID: 2C526530-CD5A-5D50-AE41-6199FE23C999; **Taxon:** scientificName: Californiphilus hubeiensis; **Location:** country: China; stateProvince: Hubei; county: Jingshan; locality: Taizishan National Forest Park; verbatimElevation: 140 m; verbatimCoordinates: 30.9300°N, 112.9300°E; **Event:** eventDate: 2.iv.2019; **Record Level:** language: en; institutionCode: CMMI
- **Type status:** Paratype. **Occurrence:** catalogNumber: 20210410126; recordedBy: Chao Jiang; individualCount: 1; sex: male; lifeStage: adult; occurrenceID: 164D8651-66CA-5008-88BC-E6D2B6C46248; **Taxon:** scientificName: Californiphilus hubeiensis; **Location:** country: China; stateProvince: Hubei; locality: Mt. Huzhuashan; verbatimElevation: 204 m; verbatimCoordinates: 31.0774°N, 112.8928°E; **Event:** eventDate: 10.iv.2021; **Record Level:** language: en; institutionCode: CMMI

#### Description

**General features**. Body length of males 47–52 mm, with 73–79 (males) leg-bearing segments. Body narrowing forwards and towards posterior tip. Colour (in ethanol 75%) yellow; forcipules darker.

**Figure 2.**
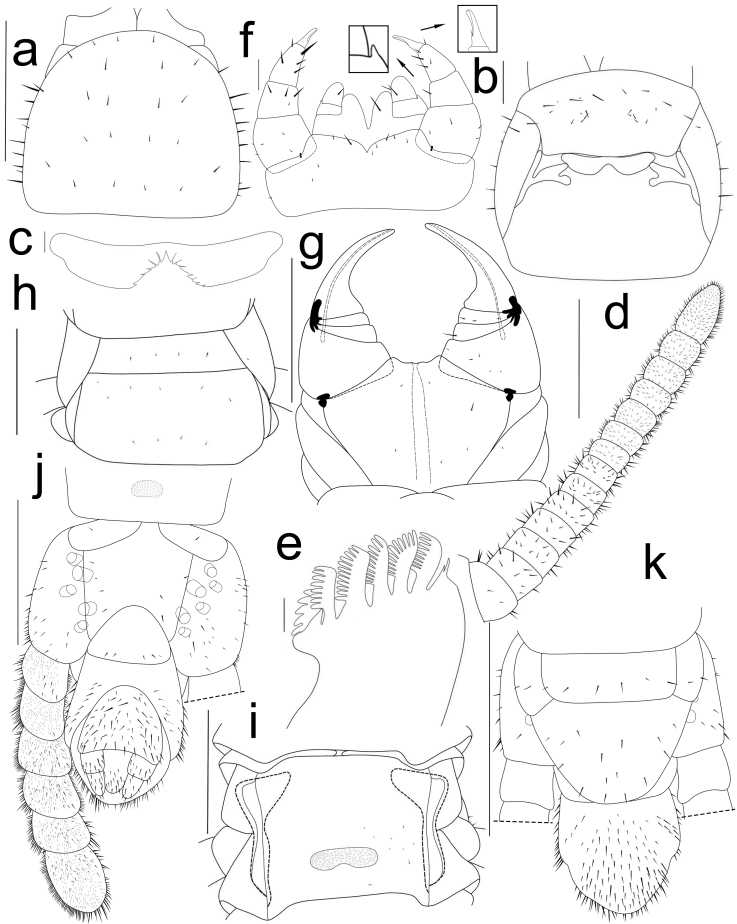
*Californiphilus hubeiensis* Yang, You & Jiang, sp. nov. **a** Cephalic capsule, dorsal; **b** Anterior part of cephalic capsule, ventral (forcipules, maxillae and mandibles removed); **c** Labrum, ventral view; **d** Right antenna, ventral; **e** Mandible; **f** Maxillary complex, ventral; **g** Forcipular segment; **h** Tergite of leg-bearing segment, dorsal; **i** Sternum of the middle trunk segments, ventral (setae on right part omitted); **j** Posterior end of body in adult male, dorsal (ultimate leg omitted); **k** Posterior end of body in adult male, ventral (Left leg omitted), ventral. Specimen: male, holotype (**a–k**). Scale bars: 250 μm (**a, d, g–k**); 100 μm (**b, f**); 20 μm (**c, e**).

**Cephalic capsule** (Fig. 2a) widest caudad, being a little narrowed cephalic to anterior corners, the border in front of corners triangular, ca. 1.2× as wide as long; lateral margins slightly convex and converging forwards, posterior margin almost straight; transverse suture absent; setae randomly scattered.

**Clypeus and labrum** (Fig. 2b and c) Clypeus anterior part with a few randomly scattered setae, 1+1 post-antennal setae aligned in five transverse rows on the anterior part of the clypeus, the first row bearing ca. 3 setae, the second row ca. 15 setae, the third row ca. 15 setae, fourth row ca. 7 setae and fifth row ca. 5 setae. Without areolate part. Labrum median arc deeply embayed, with 7 denticles on each side; entirely demarcated from clypeus by clypeolabral suture.

**Antennae** (Fig. 2d) composed of 14 articles, attenuated distally. Basal articles 1–4 slightly wider than long or sub-quadrate, with sparse and long setae. From ca. 7 article onwards, setae much shorter and denser. Ultimate article distinctly elongated, ca. 1.6× as long as wide and 2× length of penultimate article.

**Mandible** (Fig. 2e) with a deeply pigmented strong dentate lamella, long axis slightly angled to distal mandibular edge, occupying ca. 1/4 width of distal edge, with five well-developed pectinate lamellae, a rudiment pectinate lamella. Pectinate lamella each with 10–16 comb teeth, dentate lamella with 6–7 denticules.

**First maxillae** (Fig. 2f) coxosternite of first maxillae divided; telopodite biarticulated, with the basal article cylindrical and the distal article conical; telopodital lappet present, short and pointed, ca. 1/10 the length of the basal article. Each side of distal article with 3 setae apically.

**Second maxillae** (Fig. 2f) coxosternite with a slight median notch, anterior part with sparse scattered setae. Telopodite three-segmented: article 1 with short and sparse setae scattered on the outer margin; article 2 and article 3 with longer setae concentrated on the inner margin and the anterior part. Claws slightly spatulate, without basal spine.

**Forcipular segment** (Fig. 2g) forcipular tergite sub-trapezoid; ca. 2.8× as wide as long. Coxosternite exposed part ca. 1.6× as wide as long, trochanteroprefemur ca. 1.3× as wide as long; nterior margin moderately projecting with respect to its condyles; anterior border approximately straight medially; coxopleural sutures strongly converging backwards; chitin-lines complete, reaching the coxosternal condyle. Tarsungulum large and pointed, not reaching cephalic capsule anterior margin when closed. All articles without denticles. Poison gland calyx linear, ca. 6.0× as long as wide, situated in anterior 1/4 of trochanteroprefemur. Entire forcipular segment without denticles.

**Leg-bearing segment** (Fig. 2h and i) Tergites sub-trapezoid, ca. 2.4× as wide as long, smooth, without paramedian sulci and preparatergites. Metasternites sub-trapezoid, ca. 1.1× as wide as long, with short sparse scattered setae. Lateral gutter and virguliform fossa begin on segments 26–29 and extend to segments 36–40. Posterior reniform ventral pore-fields present in all sternites from 1 to penultimate, pore-fields posteriorly slightly embayed. Pore-fields on sternite 1 distinctly smaller than those on succeeding sternites, sub-oval. Penultimate sternite posterior and lateral margins nearly straight; pore-fields transversally extending over 2/3 of sternite width and covering 1/2 of sternite length.

**Ultimate leg-bearing segment** (Fig. 2j and k) Pretergite ca. 3.4× as wide as long on exposed part. Metatergite ca. 1.5× as long as wide; sub-trapezoid. converging backwards; posterior margin ca. 0.7× as wide as anterior margin; with sparse setae of various sizes. Metasternite sub-trapeziform, ca. 1.2× as long as wide; narrowing posteriorly, posterior margin concave anteriorly, without median sulci; coxal pores 20–26 on each side, opening inside ventral and dorsal pouches.

Ultimate leg ca. 1.1× as long as penultimate leg, without claw; male distinctly swollen, ventral and lateral sides with dense setae, female short and slender, with sparse setae.

**Postpedal segments** (Fig. 2j and k) Male gonopods bi-articulate, with dense setae; penis conical. Anal pores absent.

#### Diagnosis

Body length reaching at least 47 mm; number of leg-bearing segments usually 73–79; Lateral gutter and virguliform fossa begin on segments 26–29 and extend to segments 37–40. Penultimate ventral pore-fields transversally extending over 2/3 of sternal width and covering 1/2 of sternal length. Ultimate leg-bearing segment: Coxal pores numerous, clustered into two dorsal and usually two ventral common pouches.

#### Etymology

The specific epithet refers to its distribution, Hubei Province. We suggest its Chinese common name as “湖北地蜈蚣”.

#### Distribution

*C. hubeiensis* is known from four localities in Hubei, China (Ma'anshan in Wuhan, Tonghu Wetland Park, Taizishan Forest Park in Jingshan County and Mt. Huzhuashan), all in the Dabie Mountain range and adjacent areas, within subtropical evergreen broad-leaved forests, mainly in secondary forest patches in parks.

#### Notes

The present species closely resembles *Californiphilus japonicus* in morphology, sharing several features: the telopodite of the first maxillae with telopodital lappets, presence of a lateral gutter and a virguliform fossa and coxal pores opening inside ventral and dorsal pouches. However, the two species differ in the size and shape of the ventral pore fields on the penultimate sternite. In the present species, the penultimate ventral pore fields are large, transversely extending over 2/3 of the sternal width and covering half of the sternal length. Whereas in *C. japonicus*, according to Takakuwa (1935), sternites from the first to the penultimate bear a kidney-shaped ventral pore field, but no enlargement of these fields on the penultimate sternite was mentioned in that description.

This species is morphologically similar to *C. michelbacheri* and both have an enlarged penultimate sternite. However, *C. michelbacheri* has more than 120 leg-bearing segments and the pore fields on the last few sternites are all enlarged (Attems 1947), whereas the present species has 73–79 leg-bearing segments and only the penultimate pore field is enlarged, making them easy to distinguish.

We noticed that all specimens possessing an enlarged ventral pore field on the penultimate sternite are male. This suggests a possible case of sexual dimorphism, where the pore field is expanded in males, but remains in the sub-reniform shape typical of the genus *Californiphilus* in females. This hypothesis could be tested in future studies by increasing the sample size.

### Californiphilus nanus

Yang, You & Jiang sp. nov.

8B61EA98-A4F1-59D2-ADFC-0BCC7946B733

438F3394-8F8D-4692-BFCC-16C571751BFB

#### Materials

- **Type status:** Holotype. **Occurrence:** catalogNumber: 20250413013; recordedBy: Yuan Xiong& Jing Zhong; individualCount: 1; sex: male; lifeStage: adult; occurrenceID: 1C64CC4D-ADCA-55D9-B157-199F79C3A077; **Taxon:** scientificName: Californiphilus nanus; **Location:** country: China; stateProvince: Huna; locality: Hefu National Forest Park; verbatimElevation: 85 m; verbatimCoordinates: 29.0548°N, 111.6022°E; **Event:** eventDate: 13.iv.2025; **Record Level:** language: en; institutionCode: CMMI
- **Type status:** Paratype. **Occurrence:** catalogNumber: 20240728006D; recordedBy: Huiqin Ma; individualCount: 1; sex: male; lifeStage: adult; occurrenceID: 2E61EA4E-7B24-5167-A504-FE3F00A68452; **Taxon:** scientificName: Californiphilus nanus; **Location:** country: China; stateProvince: Chongqing; county: Wuxi; locality: Yintiaoling Nature Reserve Guan Mountain Protection Area; verbatimElevation: 1290 m; verbatimCoordinates: 31.4747°N, 109.8646°E; **Event:** eventDate: 28.vii.2024; **Record Level:** language: en; institutionCode: CMMI
- **Type status:** Paratype. **Occurrence:** catalogNumber: 20250507014D; recordedBy: Yifei Yu; individualCount: 1; sex: female; lifeStage: adult; occurrenceID: FD3044B5-5C21-5D54-98AD-70FFDD5B9F56; **Taxon:** scientificName: Californiphilus nanus; **Location:** country: China; stateProvince: Chongqing; county: Wuxi; locality: Yintiaoling Nature Reserve Linkouzi Protection Area; verbatimElevation: 1290 m; verbatimCoordinates: 31.4747°N, 109.8646°E; **Event:** eventDate: 7.v.2025; **Record Level:** language: en; institutionCode: CMMI
- **Type status:** Paratype. **Occurrence:** catalogNumber: 20250513025D; recordedBy: Yutong Zhang & Hongyan Zhang; individualCount: 1; sex: female; lifeStage: adult; occurrenceID: 1EDD3EBE-1E99-5D75-9F46-D73705161333; **Taxon:** scientificName: Californiphilus nanus; **Location:** country: China; stateProvince: Chongqing; county: Wuxi; locality: Yintiaoling Nature Reserve Yinglan Protection Area; verbatimElevation: 940 m; verbatimCoordinates: 31.2627°N, 109.5141°E; **Event:** eventDate: 13.v.2025; **Record Level:** language: en; institutionCode: CMMI
- **Type status:** Paratype. **Occurrence:** catalogNumber: 20250328050; recordedBy: Chao Jiang& Jing Zhong; individualCount: 1; sex: female; lifeStage: adult; occurrenceID: 89F708AB-7FAC-5F76-A2CC-499F1259F61C; **Taxon:** scientificName: Californiphilus nanus; **Location:** country: China; stateProvince: Hubei; locality: Kongshandong Cave Scenic Area; verbatimElevation: 186 m; verbatimCoordinates: 30.9734°N, 113.0376°E; **Event:** eventDate: 28.iii.2025; **Record Level:** language: en; institutionCode: CMMI

#### Description

**General features**. Body length of males 23–42 mm, of females 16–20 mm, with 51–57 (females: 51; males: 57) leg-bearing segments. Body narrowing forwards and towards posterior tip. Colour (in ethanol 75%) light yellow; forcipules darker.

**Figure 3.**
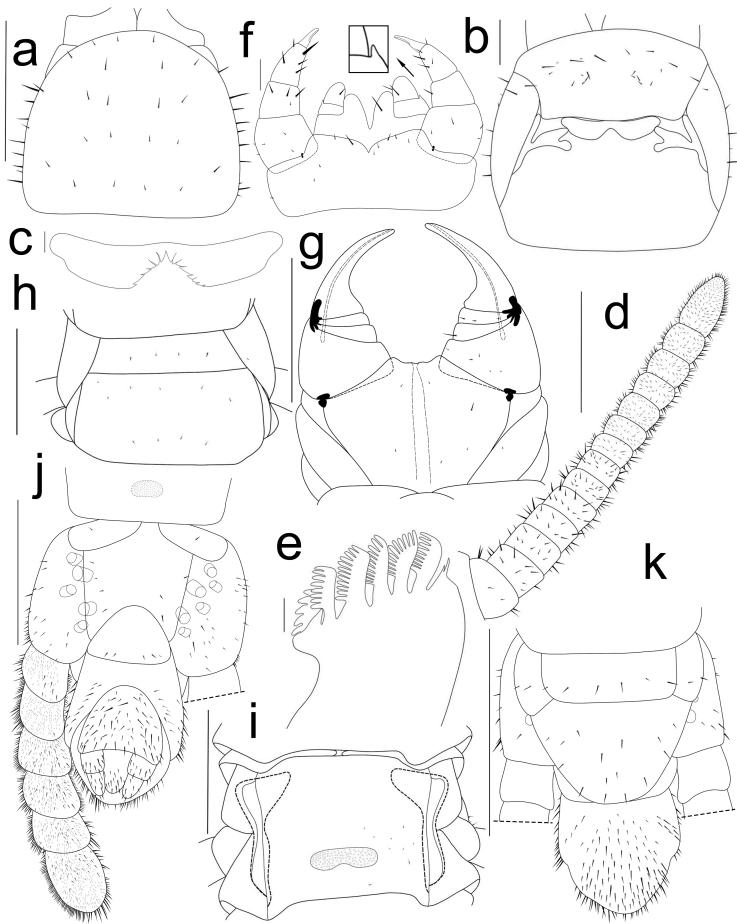
*Californiphilus nanus* Yang, You & Jiang, sp. nov. **a** Cephalic capsule, dorsal; **b** Anterior part of cephalic capsule, ventral (forcipules, maxillae and mandibles removed); **c** Labrum, ventral view; **d** Right antenna, ventral; **e** Mandible; **f** Maxillary complex, ventral; **g** Forcipular segment; **h** Tergite of leg-bearing segment, dorsal; **i** Sternum of the middle trunk segments, ventral (setae on left part omitted); **j** Posterior end of body in adult male, dorsal (ultimate leg omitted); **k** Posterior end of body in adult male, ventral (left leg omitted), ventral. Specimen: male, holotype (**a–k**). Scale bars: 500 μm (**a, d, g–h, j, k**); 100 μm (**b**); 50 μm (**f**); 20 μm (**c, e**).

**Cephalic capsule** (Fig. 3a) Sub-trapezoid; ca. 1.2× as wide as long; lateral margins slightly convex converging forwards, posterior margin almost straight; transverse suture absent, long setae arranged scattered.

**Clypeus and labrum** (Fig. 3b and c) Clypeus anterior part with a few randomly scattered setae, 1+1 post-antennal setae aligned in two transverse rows on the anterior part of the clypeus, the first row bearing ca. 6 setae and the second row ca. 3 setae. Without areolate part. Labrum median arc deeply embayed, with 6–7 denticles on each side; entirely demarcated from clypeus by clypeolabral suture.

**Antennae** (Fig. 3d) composed of 14 articles, attenuated distally. Basal articles 1–4 slightly wider than long or sub-quadrate, with sparse and long setae. From ca. 8 article onwards, setae much shorter and denser. Ultimate article distinctly elongate, ca. 1.6× as long as wide and 2× length of penultimate article.

**Mandible** (Fig. 3e) with a deeply pigmented strong dentate lamella, long axis slightly angled to distal mandibular edge, occupying ca. 1/4 width of distal edge, with five well-developed pectinate lamellae, a rudiment pectinate lamella. Pectinate lamellae each with 8–12 comb teeth, dentate lamella with 5–6 denticules.

**First maxillae** (Fig. 3f) coxosternite of first maxillae divided; telopodite biarticulated, with the basal article cylindrical and the distal article conical; telopodital lappet present, short and pointed, ca. 1/7 the length of the basal article. Coxal process with 1 setae apically and the distal article of the telopodite with 1–2 setae each side on its anterior part.

**Second maxillae** (Fig. 3f) coxosternite with a slight median notch, anterior part with sparse scattered setae. Telopodite three-segmented: article 1 with short and sparse setae scattered on the outer margin; article 2 and article 3 with longer setae concentrated on the inner margin and the anterior part. Claws slightly spatulate, with one small basal spine.

**Forcipular segment** (Fig. 3g) forcipular tergite sub-trapezoid; ca. 2.8× as wide as long. Coxosternite exposed part ca. 1.8× as wide as long, trochanteroprefemur ca. 1.2× as wide as long; interior margin moderately projecting with respect to its condyles; anterior border approximately straight medially; coxopleural sutures strongly converging backwards; chitin-lines complete, reaching the coxosternal condyle. Tarsungulum large and pointed, not reaching cephalic capsule anterior margin when closed. All articles without denticles. Poison gland calyx linear, ca. 6.0× as long as wide, situated in anterior 1/4 of trochanteroprefemur. Entire forcipular segment without denticles.

**Leg-bearing segment** (Fig. 3h and i) Tergites sub-trapezoid, ca. 2.3× as wide as long, with short sparse setae, without paramedian sulci and preparatergites. Metasternites sub-trapezoid, ca. 1.2× as wide as long, with short sparse scattered setae. Lateral gutter and virguliform fossa begin on segments 17–19 and extend to segments 23–28. Posterior reniform ventral pore-fields present in all sternites from 1 to penultimate, pore-fields posteriorly slightly embayed. Pore-field on sternite 1 distinctly smaller than those on succeeding sternites, sub-oval.

**Ultimate leg-bearing segment** (Fig. 3j and k) Pretergite ca. 2.7× as wide as long on exposed part. Metatergite ca. 1.4× as long as wide; sub-trapezoid. converging backwards; posterior margin ca. 0.4× as wide as anterior margin; with sparse setae of various sizes. Metasternite sub-trapeziform, ca. 1.5× as long as wide; narrowing posteriorly; posterior margin concave anteriorly, without median sulci; coxal pores 4–13 on each side, opening inside dorsal and ventral pouches.

Ultimate leg ca. 1.1× as long as penultimate leg, without claw; male distinctly swollen, ventral and lateral sides with dense setae, female short and slender, with sparse setae.

**Postpedal segments** (Fig. 3j and k) Female gonopods unarticulated, posterior margin slightly concave, uniformly with sparse setae; male gonopods bi-articulate, with dense setae; penis conical. Anal pores absent.

#### Diagnosis

Body length reaching at least 16 mm; number of leg-bearing segments usually 51–57; The second maxillary claw with one basal spine. The legs possess remarkably long setae. The body length is relatively short and the segments with lateral gutter and virguliform fossa are located more anteriorly compared to *Californiphilus japonicus* (Takakuwa, 1935), beginning on segments 17–19 and extending to segments 23–28. The diameter of the coxal pores is larger than that of the known species, while the quantity is fewer, not exceeding 13 on each side.

#### Etymology

Latin: *nanus* = dwarf / small. The specific epithet refers to its relatively small body size compared to that of *Californiphilus japonicus* (Takakuwa, 1935). We suggest its Chinese common name as “小型加州地蜈蚣”.

#### Distribution

*C. nanus* is known from five localities in Hunan, Chongqing and Hubei, China (Hefu Forest Park in Hunan; three sites in Yintiaoling Nature Reserve, Wuxi County, Chongqing: Guan Mountain, Linkouzi and Yinglan; and Kongshandong Scenic Area in Hubei), all in the Wuling Mountain range and adjacent areas, within subtropical evergreen broad-leaved forests, mainly in secondary forest patches in reserves and parks.

#### Notes

This species differs from other *Californiphilus* species in its body size, number of segments, the position of the lateral sulci and the shape and number of the coxal pores. The body length of the examined specimens reaches at least 42 mm (based on five specimens), suggesting that the species may attain a larger size than currently documented, with no more than 60 leg-bearing segments. Lateral gutter and virguliform fossa begin on segments 17–19 and extend to segments 23–28. The coxal pores are relatively large and few in number, only 4–13, whereas other species possess no fewer than 20, sometimes exceeding 40.

**Comparison with *Californiphilus japonicus***. In *C. japonicus*, the body length is less than 55 mm and the number of leg-bearing segments ranges from 67 to 78, which is significantly greater than that of *C. nanus*. The anterior boundaries of the lateral gutter and virguliform fossa are located much more posteriorly compared to *C. nanus* (see Table 1). While *C. nanus* bears relatively long setae on its walking legs, this character is not specified in the literature or illustrations of *C. japonicus*. Furthermore, *C. japonicus* possesses a larger number of coxal pores with slightly smaller diameters, whereas *C. nanus* has fewer, but larger coxal pores.

Although morphologically similar to *Californiphilus japonicus*, the present species does not represent small-sized individuals of the latter. Despite their smaller body size, the examined specimens (CMMI 20250413013, 20240728006D, 20250507014D) possess fully developed gonopods. Detailed comparisons with *C. japonicus* of equivalent body size confirm that the aforementioned diagnostic characters remain stable.

**Figure 4.**
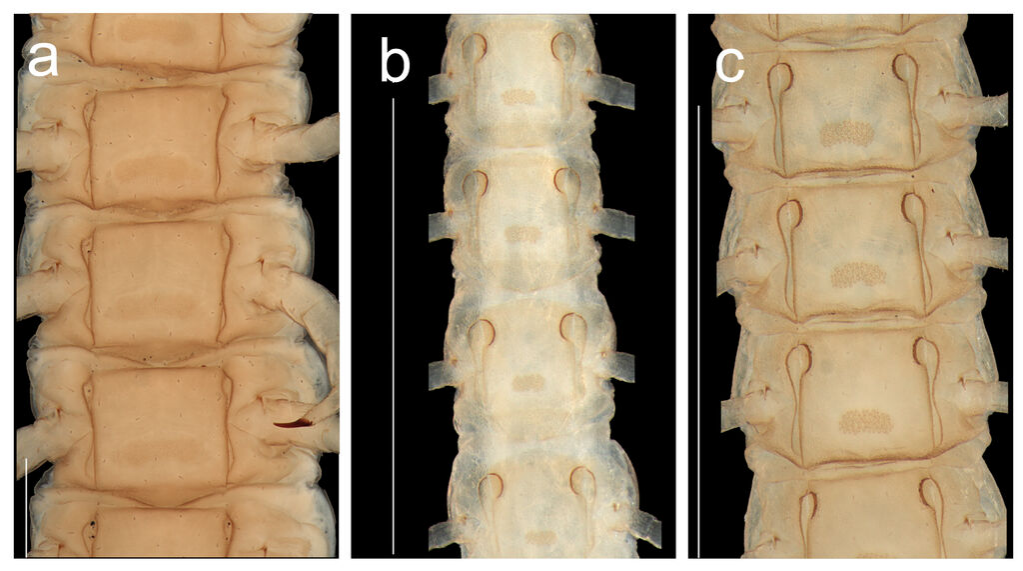
*Californiphilus nanus* Yang, You & Jiang, sp. nov. Sternum of the middle trunk segments, ventral. **a** spm. CMMI 20240728006D; **b** spm. CMMI 20250328050; **c** spm. CMMI 20250507014D. Scale bars: 1 mm (**a–c**).

This species presents considerable intraspecific variation in the shape of the lateral gutter and virguliform fossa (Leśniewska et al. 2009). In some individuals (observed in males), the virguliform fossa may appear subtriangular (Fig. 4a and Fig. 5e). In others, the virguliform fossa forms a minute arc along with a relatively narrower lateral gutter (Fig. 4a). Female specimens exhibit these structures as semicircular (Fig. 4b and c). Adult female (CMMI 20250328050, 20250513025D) typically exhibit a semicircular shape (Fig. 4b).

## Identification Keys

### Key to species of the genus *Californiphilus*

**Table:** 

1	Labrum median arc medial part with denticle	2
–	Labrum median arc medial part absent denticle, each side with 4–5 denticles	*C. mexicanus*
2	Mandible with 5–7 pectinate lamellae, trunk with less than 100 pairs of legs	3
–	Mandible with 4 pectinate lamellae, trunk with 129 pairs of legs (Attems 1947)	*C. michelbacheri*
3	Coxal pores opening underside ventral and dorsal pouches	4
–	Coxal pores opening beneath metasternite and metatergite of the ultimate leg-bearing segment	*C. cryptoporus* sp. nov.
4	Ventral pore-fields on the penultimate sternite are not enlarged, being similar in size to those on the preceding segments	5
–	Penultimate ventral pore fields large, transversally extending over 2/3 of sternal width and covering 1/2 of sternal length	*C. hubeiensis* sp. nov.
5	The lateral gutter and virguliform fossa begin on segments 25–30 and extend to segments 34–40, body with 67–78 pairs of legs, 16 or more coxal pores on each side	*C. japonicus*
–	The lateral gutter and virguliform fossa begin on segments 17–19 and extend to segments 23–28, body with 51–57 pairs of legs; 13 or less coxal pores on each side; second maxillary claw with one basal spine	*C. nanus* sp. nov.

## Discussion

The three new species are referred to *Californiphilus*, based on a combination of diagnostic features shared with its congeners and absent in all other himantariids: V‑shaped anterior notch of the second maxillary coxosternite, absence of paratergites and transverse sternal grooves, transversely elongated sternal pore‑fields on most segments, coxal pores opening into dorsal and ventral pouches, trapezoidal ultimate metasternite with emarginate posterior margin, ultimate legs without a pretarsus and smooth forcipular tarsungulum lacking denticle. This combination clearly separates the genus from its relatives— for example, coxal pores are absent in Mesocanthus, restricted dorsally in *Polyporogaster* and superficial in *Himantarium* and *Stigmatogaster*, while *Bothriogaster* possesses horseshoe‑shaped sternal depressions (Attems 1929; Bonato et al. 2011). The generic placement is therefore well justified.

**Figure 7.**
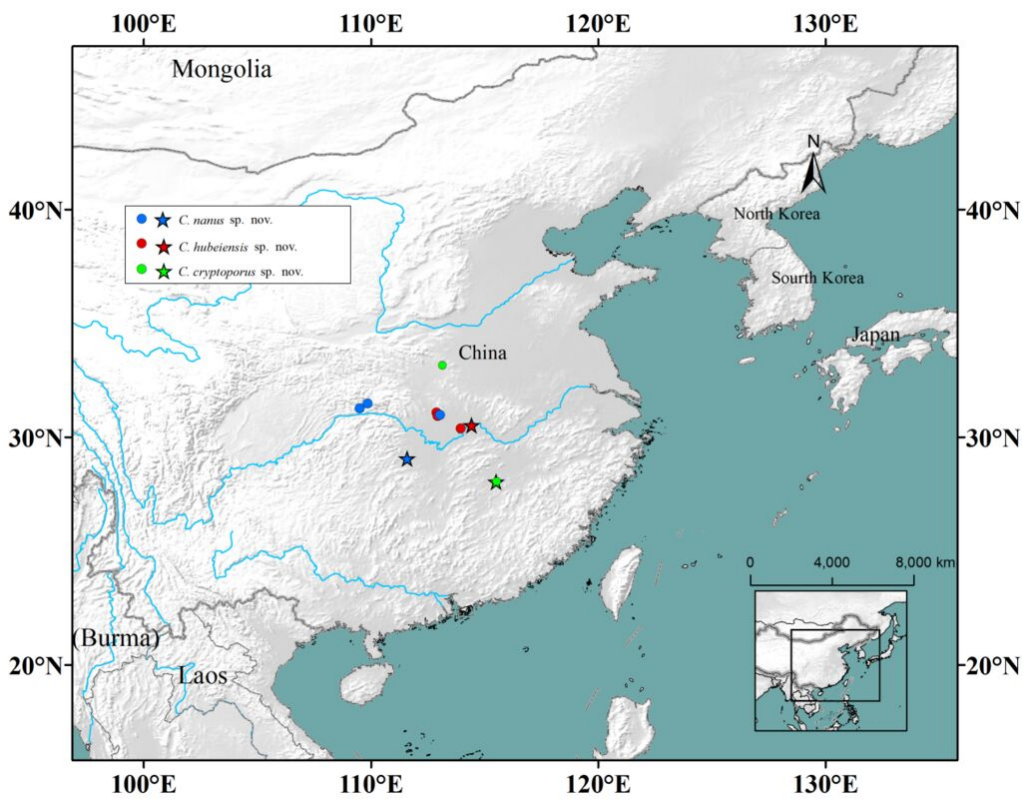
Collected localities *Californiphilus* in this study. Stars represent type localities.

In this study, we describe three new species of genus Californiphilus, all of which are distributed in the middle and lower reaches of the Yangtze River (Fig. 7). Notably, *C. nanus* and *C. hubeiensis* are located within the recently identified Global Biodiversity Hotspot: the Central China Hotspot (Feng et al. 2026). For *C. nanus*, the observed pattern of males having more leg-bearing segments than females is based on limited material and may reflect sampling bias, as females with higher counts may not have been collected. Although only 7% of primary natural vegetation remains in this hotspot, it harbours a high concentration of endemic species. These three new species primarily inhabit secondary forests in urban and forest parks, consistent with the Central China Hotspot, suggesting that many more geophilomorph species in this region remain to be discovered.

The three species differ slightly in body size, a pattern that may be linked to the region's topographic complexity (Table 1). The fragmented topography of central China has dramatic altitudinal fluctuations and complex variations in slope aspect and gradient. Different microhabitats, such as deep valleys, steep slopes and mountain ridges, are spatially separated. These factors likely cause the observed body size differences. Such morphological divergence within a limited geographic range suggests that local topographic heterogeneity may promote subtle evolutionary differentiation, even amongst closely related species（Ye et al. 2021）.

## Supplementary Material

XML Treatment for Californiphilus cryptoporus

XML Treatment for Californiphilus hubeiensis

XML Treatment for Californiphilus nanus

## Figures and Tables

**Figure 5a.**
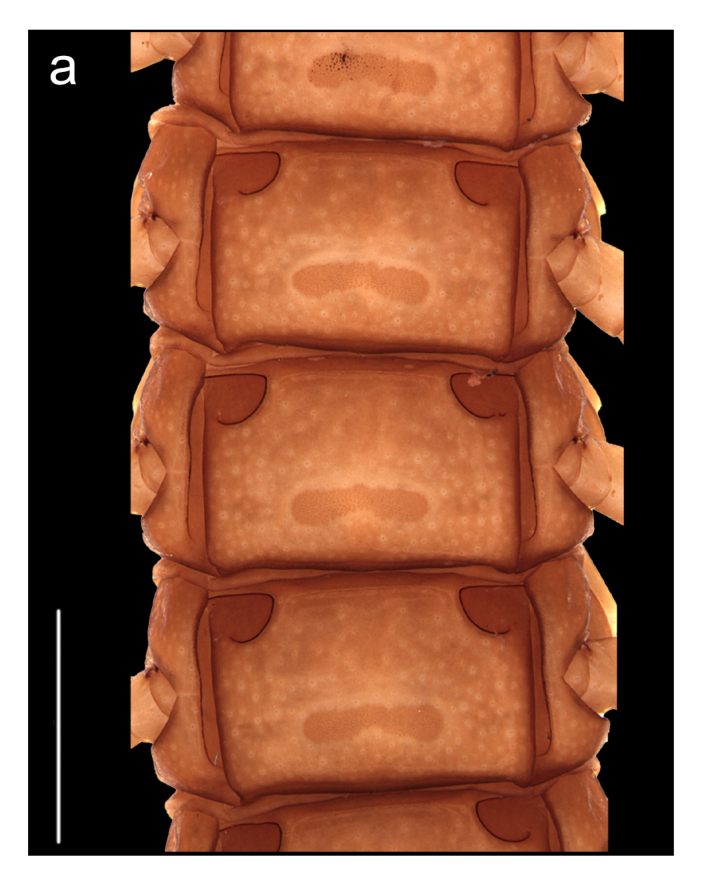
*C. cryptoporus* sp. nov., holotype (spm. CMMI 20231021005D);

**Figure 5b.**
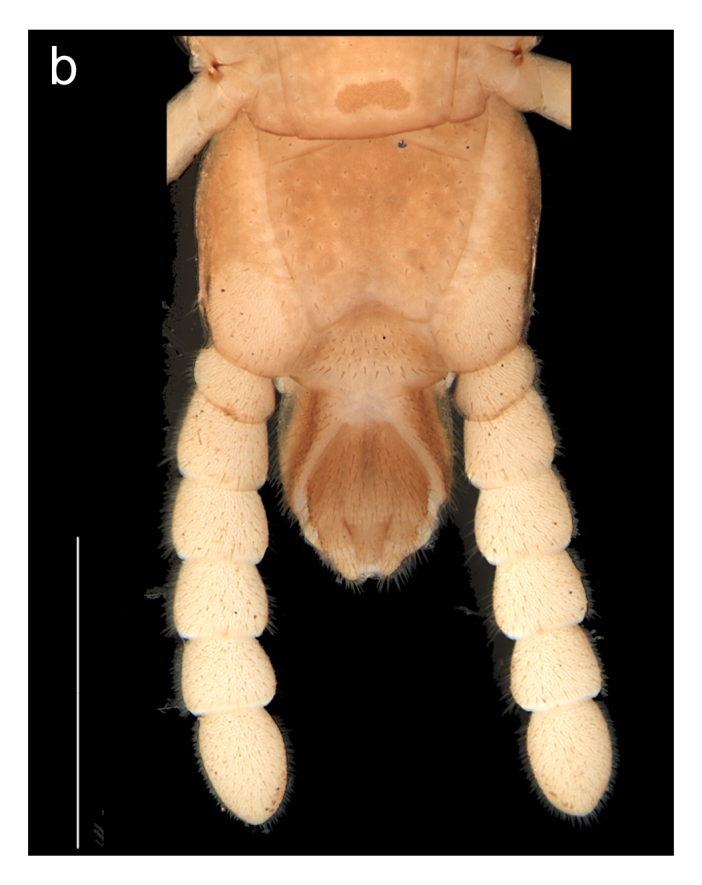
*C. cryptoporus* sp. nov., holotype (spm. CMMI 20231021005D);

**Figure 5c.**
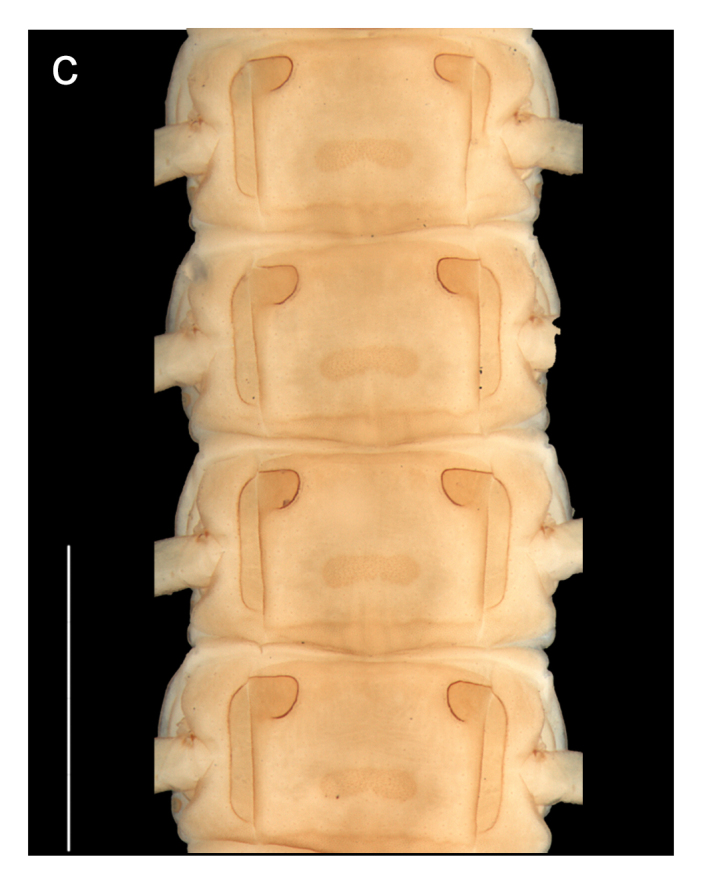
*C. hubeiensis* sp. nov., holotype (spm. CMMI 20230427003D);

**Figure 5d.**
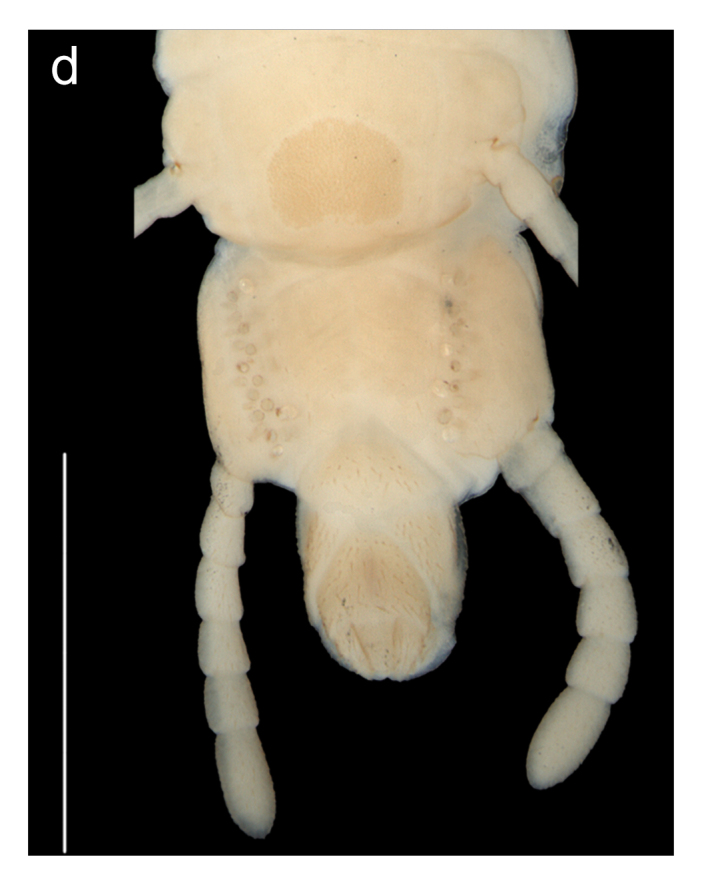
*C. hubeiensis* sp. nov., holotype (spm. CMMI 20230427003D);

**Figure 5e.**
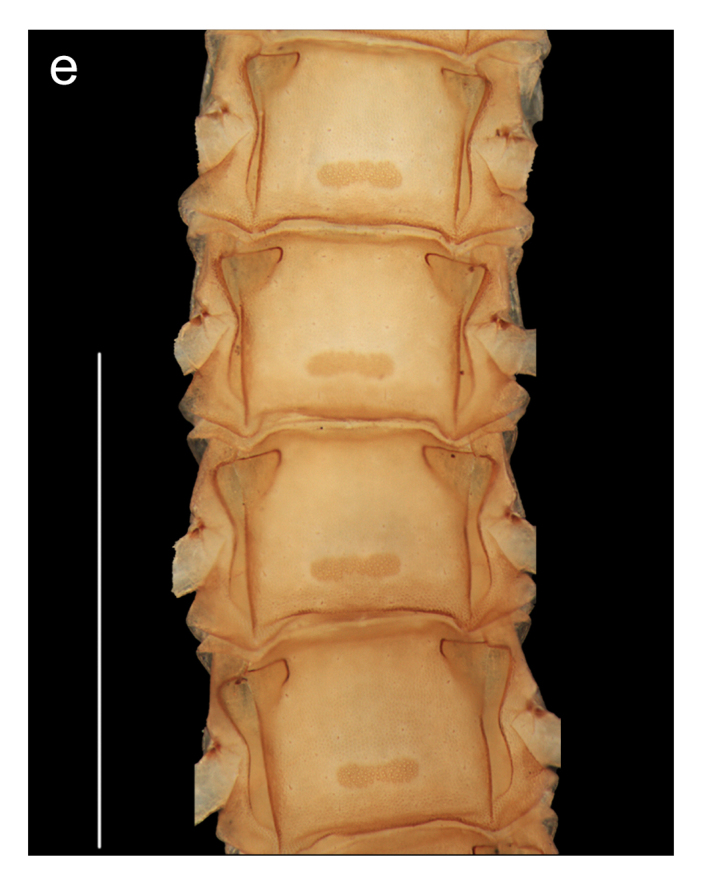
*C. nanus* sp. nov., holotype (spm. CMMI 20250413013);

**Figure 5f.**
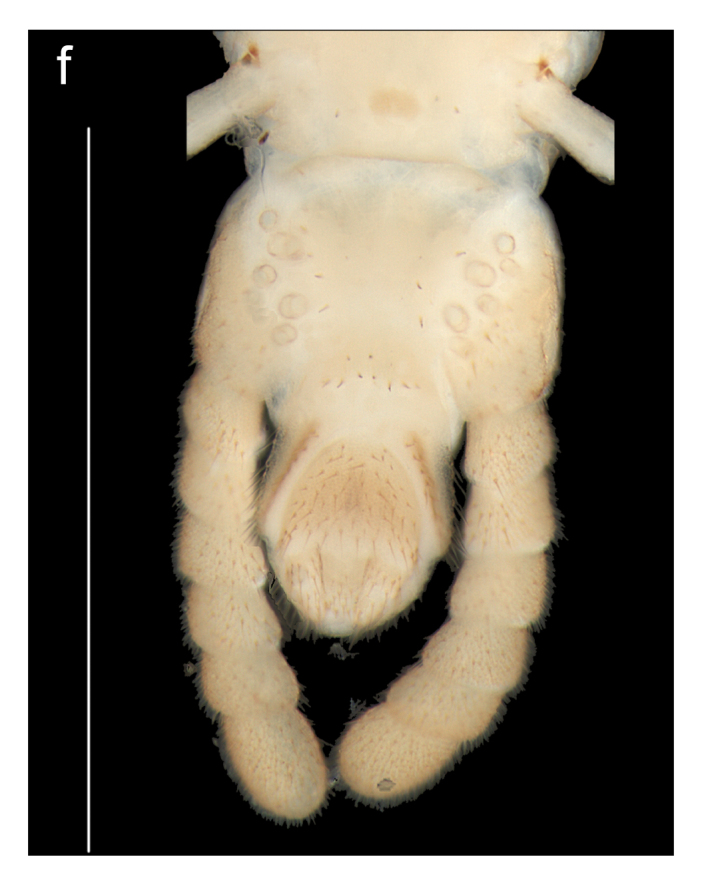
*C. nanus* sp. nov., holotype (spm. CMMI 20250413013).

**Figure 6a.**
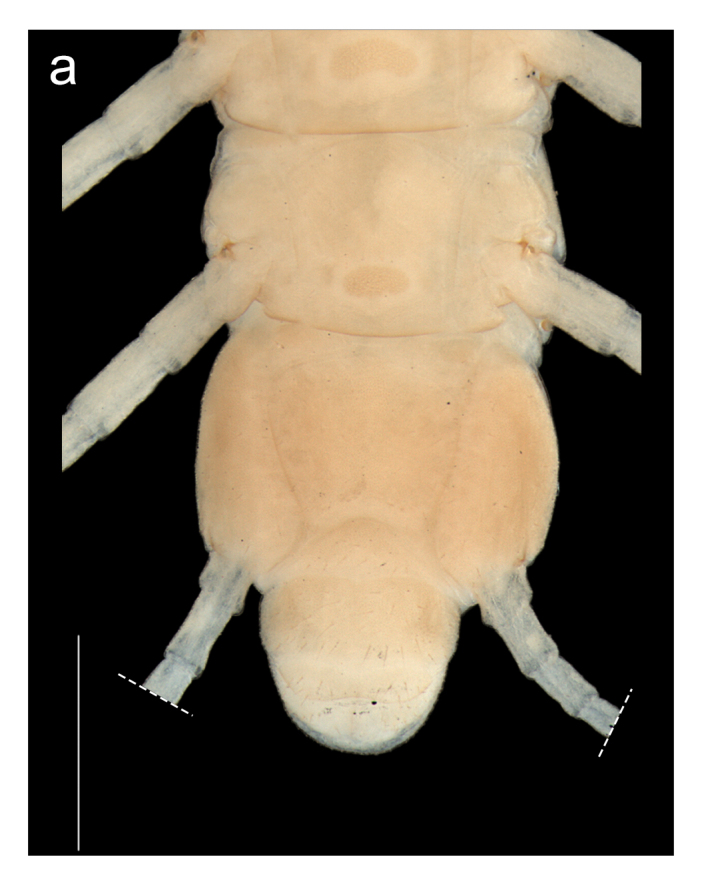
*C. cryptoporus* sp. nov., holotype (spm. CMMI 20191016030 );

**Figure 6b.**
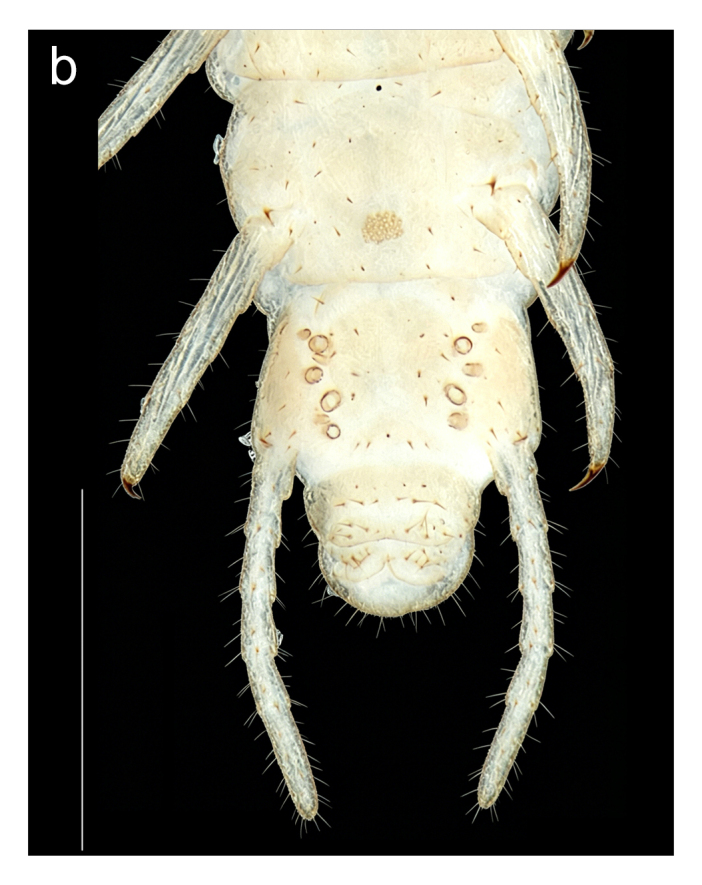
*C. nanus* sp. nov., holotype (spm. CMMI 20250507014D).

**Table 1.** Main morphological differences amongst all known species of the genus *Californiphilus*. Data after Takakuwa 1935, Takakuwa 1940, Attems 1947, Bonato et al. 2007 and Bonato et al. 2011 (n = number of examined specimens).

Characters		*C. cryptoporus* sp. nov (n = 7)	*C. hubeiensis* sp. nov (n = 4)	*C. nanus* sp. nov (n = 5)	*C. japonicus* (Takakuwa 1935)	*C. mexicanus* (Attems 1947)	*C. michelbacheri* (Verhoeff 1938)
Maximum observed body length (mm)		≥ 63 mm	≥ 52 mm	≥ 42 mm	shorter than 55 mm	/	56
Number of leg pairs		71–77	73–79	51–57	67–78	ca. 145	ca. 129
Position of lateral gutter and virguliform		Begin on segments 23–26 and extend to segments 34–39	Begin on segments 26–29 and extend to segments 37–40	Begin on segments 17–19 and extend to segments 23–28	Begin on segments 25–30 and extend to segments 34–40	/	/
Labrum median arc denticle		8+ 10	7+ 7	6+ 7	ca. 10	Median arc medial part absent denticle, each side with 4–5 denticle	With a deep median arc and 4 small teeth on each side
Mandible	well-developed pectinate lamellae	7	5	5	4–5	3	4
	pectinate lamella comb	6–14	10–16	8–12	8–16	/	/
	dentate lamella teeth	6–7	6–7	5–6	6–7	11	11
Coxal pores		Opening underside of the metasternite of the ultimate leg-bearing segment; 28–30 on each side	Opening inside ventral and dorsal pouches; 20–26 on each side	Opening inside ventral and dorsal pouches; 4–13 on each ventral side	Opening inside ventral and dorsal pouches	Opening inside ventral and dorsal pouches	Opening underside of the metasternite of the ultimate leg-bearing segment; in 3 tufts (1 dorsal largest under lateral tergite & intercalary; 2 ventral smaller over sternite sides)
Pore-fields	Pore-field distribution and shape	Sternites 1st to penultimate sternites with a kidney‑shaped ventral pore field	Sternites 1st to antepenultimate with a kidney‑shaped ventral pore field	Sternites 1st to penultimate sternites with a kidney‑shaped ventral pore field	Sternites 1st to penultimate sternites with a kidney‑shaped ventral pore field	Sternites 2nd to penultimate: pore-field sharply delimited, much wider than long, posteriorly shallowly emarginate, laterally rounded	Sternites 2nd to 38th with transverse pore-fields, with penultimate typical, 3rd–6th from last enlarged, 7th from last similar, and 8th–12th from last reduced to minimum at 12th
	Penultimate ventral pore-fields	Sub-reniform, the same size as those on the preceding segments	Large, transversally extending over 2/3 of sternal width and covering 1/2 of sternal length	Sub-reniform, the same size as those on the preceding segments	Sub-reniform	Shape as in preceding pore-fields; size not decreasing caudally	transverse pore-field, without obvious size variation
